# A process evaluation, with mediation analysis, of a web-based intervention to augment primary care exercise referral schemes: the e-coachER randomised controlled trial

**DOI:** 10.1186/s12966-022-01360-7

**Published:** 2022-09-29

**Authors:** Jeffrey Lambert, Adrian Taylor, Adam Streeter, Colin Greaves, Wendy M. Ingram, Sarah Dean, Kate Jolly, Nanette Mutrie, Rod S. Taylor, Lucy Yardley, Lisa Price, John Campbell

**Affiliations:** 1 Department for Health, University of Bath, Bath, UK; 2 Peninsula Medical School (Faculty of Health), University of Plymouth, Plymouth, UK; 3 Institute for Epidemiology and Social Medicine, Muenster University, Muenster, Germany; 4 School of Sport, Exercise and Rehabilitation Sciences, University of Birmingham, Birmingham, UK; 5 University of Exeter Medical School, Exeter, UK; 6 Institute of Applied Health Research, University of Birmingham, Birmingham, UK; 7 Physical Activity for Health Research Centre, University of Edinburgh, Edinburgh, UK; 8 MRC/CSO Social & Public Health Sciences Unit, University of Glasgow, Glasgow, UK; 9 School of Psychological Science, University of Bristol, Bristol, UK; 10 School of Psychology, University of Southampton, Southampton, UK; 11 Sport and Health Sciences, University of Exeter, Exeter, UK

**Keywords:** Exercise referral scheme, Physical activity, Chronic conditions, Web-based support, Self-determination theory, Accelerometer

## Abstract

**Background:**

The e-coachER trial aimed to determine whether adding web-based behavioural support to exercise referral schemes (ERS) increased long-term device-measured physical activity (PA) for patients with chronic conditions, compared to ERS alone, within a randomised controlled trial. This study explores the mechanisms of action of the e-coachER intervention using measures of the behaviour change processes integral to the intervention’s logic model.

**Methods:**

Four hundred fifty adults with obesity, diabetes, hypertension, osteoarthritis or history of depression referred to an ERS were recruited in Plymouth, Birmingham and Glasgow. The e-coachER intervention comprising 7-Steps to Health was aligned with Self-Determination Theory and mapped against evidence-based behaviour change techniques (BCTs). Participants completed questionnaires at 0, 4, and 12 months to assess PA and self-reported offline engagement with core BCTs in day-to-day life (including action planning and self-monitoring) and beliefs relating to PA (including perceived importance, confidence, competence, autonomy and support). We compared groups at 4 and 12 months, controlling for baseline measures and other covariates. Mediation analysis using the product of coefficients method was used to determine if changes in process variables mediated intervention effects on moderate to vigorous physical activity (MVPA) recorded by accelerometer and self-report at 4- and 12-months.

**Results:**

The internal reliability (Cronbach’s alpha) for all multi-item scales was > 0.77. At 4-months, those randomised to e-coachER reported higher levels of PA beliefs relating to importance (1.01, 95% confidence interval (CI): 0.42 to 1.61, *p =* 0.001), confidence (1.28, 95% CI: 0.57 to 1.98, *p <* 0.001), competence (1.61, 95% CI: .68 to 2.54, *p =* 0.001), availability of support (0.77, 95% CI: 0.07 to 1.48, *p =* 0.031), use of action planning (1.54, 95% CI: 0.23 to 2.85, *p =* 0.021) and use of self-monitoring (0.76, 95% CI: 0.19 to 1.32, *p =* 0.009) compared to ERS alone. There were no intervention effects on autonomous beliefs or perceived frequency of support, compared to ERS alone. At the 12-month follow-up, participants belief in the importance of PA was the only process measure to remain significantly higher in the e-coachER group when compared to ERS alone (0.75, 95% CI: 0.05 to 1.45). Intervention effects on perceived importance (2.52, 95% CI: 0.45 to 5.39), action planning (1.56, 95% CI: 0.10 to 3.54) and self-monitoring (1.92, 95% CI: 0.21 to 4.33) at 4-months significantly mediated change in accelerometer measured MVPA at 12-months (recorded in ≥ 10-min bouts).

**Conclusions:**

e-coachER led to some short-term changes in most process outcomes. Some of these processes also appeared to mediate e-coachER effects on changes in accelerometer measured MVPA. Further work should be carried out to understand how best to design and implement theoretically underpinned web-based physical activity promotion interventions within ERS.

**Trial registration:**

ISRCTN, ISRCTN15644451. Registered 12 February 2015.

## Background

Physical activity (PA) is an evidence-based therapy for a range of chronic physical and mental health conditions, such as obesity, hypertension, type 2 diabetes, lower limb osteoarthritis and depression [1]. Primary care exercise referral schemes (ERS) aim to facilitate the promotion of PA in non-clinical settings and are primarily delivered in leisure centres and gyms in structured programmes, though not exclusively [2]. ERS involves a patient being referred to a local physical activity specialist or service, followed by an individual assessment and physical activity programme [2]. However, ERS have only been modestly effective at increasing PA and improving health conditions [3]. A meta-analysis of eight randomised trials showed ERS led to only small increases in the proportion of participants achieving 90–150 min of moderate to vigorous-intensity physical activity (MVPA) per week, compared with no exercise control at 6–12 months follow-up [4].

Poor attendance and adherence may partially explain the limited effectiveness of ERS on MVPA. A systematic review showed that average uptake to ERS ranged from 66 to 81%, and average adherence rates (attending ≥ 75% of sessions) ranged from 43% in randomised trials to 49% in observational studies [5]. In a recent retrospective data linkage study of over 83,000 referred patients, 67% had actually attended the ERS [6]. Various determinants have been linked to patient uptake and adherence including gender, age, clinical condition, and socio-economic status [5, 6]. A systematic review of 33 qualitative studies found that inconvenient timing, cost, and location of sessions were key participant reported barriers to engagement in gym-based ERS schemes. Further barriers included an intimidating gym atmosphere, a dislike of the music and TV, and a lack of confidence in operating gym equipment [2]. While this information about operational barriers to engaging in ERS may be useful in modifying the design of ERS, and an important step in facilitating ERS attendance and physical activity behaviour change, it is also important to understand how individuals can best be supported to develop and maintain an interest in being physically active in a way that provides a sense of achievement, autonomy and connection with others. Few studies have tested the effectiveness of theoretically informed enhanced motivational interventions to increase ERS uptake and adherence and sustained changes in MVPA, especially for patients with chronic conditions.

Self-determination theory (SDT) is a theory of human motivation and posits that people are more likely to persevere and achieve their desired goals when intrinsically motivated [7]. When people feel more autonomous (having control over choices), competent (feel able to meet demands) and connected with others, they are more likely to feel intrinsically motivated [7]. SDT is well supported across a range of behaviours and has garnered increasing support in the domain of PA. For example, a systemic review showed a positive relationship between more autonomous forms of motivation and PA and wellbeing [8].

As previously mentioned, SDT has also been applied to ERS schemes with more autonomous regulations leading to positive mental health outcomes and stronger intentions to be physically active [8]. However, when comparing usual ERS with an ERS in which staff had been trained to support participants using SDT constructs, in an exploratory cluster trial, there was no difference in effects on MVPA at 3 and 6-month follow-up [9, 10]. A key reason for this lack of effect may have been due to poor intervention fidelity (i.e., the extent to which an intervention is delivered as intended) [11, 12] due to limited opportunities to train the providers in the SDT arm [9].

One way to overcome the fidelity challenge is to enhance ERS with theory-driven digital support in which a standardised intervention is easier to deliver and one can also assess which behaviour change processes are implicated in intervention effects on key outcomes. The e-coachER intervention was a web-based self-delivered programme hosted on the Lifeguide platform and designed to augment existing ERS [13]. Lifeguide is a set of open-source software tools that enables intervention designers with no experience in programming to create interactive web-based interventions to support healthy behaviour (www.LifeGuideonline.org/). Lifeguide has already been used to develop a range of public health and illness management interventions, including weight management [14], physical activity [15] and mental health support [16].

The e-coachER intervention was underpinned by SDT and targets key theoretical constructs—autonomy, competence and relatedness using evidence-based behaviour change techniques (BCTs) [17] as described in more detail elsewhere [13, 18]. Within the Logic model (Appendix 1), it was expected that e-coachER would more favourably influence some key theoretical components (i.e., a sense of competence, autonomy and relatedness, and heightened value or importance attached to the behaviour) and behaviour change processes (i.e., action planning, self-monitoring, enlisting social support) known to be involved in health behaviour change, than usual ERS.

We have previously reported that the e-coachER intervention, compared with usual ERS across 3 sites, had only a small non-significant effect on device assessed MVPA at 12 months [18, 19] and no effect on ERS uptake. The mean between-group difference (controlling for baseline and covariates) in MVPA (recorded in ≥ 10 min bouts) at 12 months was 11.8 weekly minutes (95% CI; -2.1 to 26.0, *p =* 0.10). This increased to 22.9 weekly minutes (95% CI: -3.4 to 47.8, *P =* 0.09) in favour of the ERS group, when controlling for whether participants had at least five intervention sessions in e-coachER. Although consideration of the dose of intervention did not make a difference to our conclusions, there may be a sign that engaging in the intervention logic model did have an impact on processes of change and MVPA.

The overall aim of the present manuscript was, therefore, to report on intervention effects on process measures (action planning, self-monitoring, importance, confidence, competence, autonomy and support) linked to the underpinning intervention theory and determine whether changes in these process measures mediated intervention effects on MVPA (recorded in ≥ 10 min bouts) at 12 months in line with the primary analysis. We also explored whether changes in process measures mediated intervention effects on secondary PA outcomes. Evaluating changes in processes leads to further insight into why a complex intervention either was or was not effective [20]. Also, very few studies have tested if process measures mediate intervention effects on PA, with no strong evidence that they do [21, 22]. This could be due to various methodological limitations, such as the predominant use of self-reported measures of physical activity (e.g., [23]) and a lack of statistical power to detect mediation effects in most studies conducted to date [24].

The e-coachER trial offers an opportunity to explore the mediating effects of process measures on accelerometer recorded MVPA assessed at 12-month follow-up in a large sample (*N* = 450) and add to the scarce literature in this field involving participants with chronic conditions.

The aims of this study were to:

1. Examine whether the e-coachER intervention led to favourable changes in measures of intervention processes specified by the e-coachER logic model, compared with usual ERS alone at 4 and 12 months.
2. Examine whether intervention effects on the above processes mediated the effects of the e-coachER on accelerometer-recorded MVPA (recorded in ≥ 10 min bouts) at 12 months.
3. Explore whether intervention effects on the above processes mediated the effects of the e-coachER on accelerometer-recorded MVPA (recorded in ≥ 10 min bouts) at 4-months, continuous accelerometer-recorded MVPA at 4 and 12 months and self-reported MVPA at 4 and 12 months.

## Methods

The e-coachER trial methods have been described in detail elsewhere [13, 19] but the main study characteristics are briefly outlined below.

### Population

Inactive (i.e., 0 h per week of physical exercise and in a sedentary occupation) or moderately inactive (i.e., some activity but < 1 h per week and in a sedentary occupation, or 0 h per week of physical exercise and in a standing occupation) adults according to the General Practice Physical Activity Questionnaire (GPPAQ) [25] with at least 1 chronic condition (from obesity, hypertension, type 2 diabetes, lower limb osteoarthritis and depression) in Greater Glasgow, Birmingham or Plymouth and adjacent rural areas, who had been or were about to be referred by a primary care practitioner to a local ERS were recruited between July 2015 to March 2017.

### Control

Participants in both arms of the trial were offered the usual primary care ERS.

### Intervention

Participants randomised to the intervention arm were offered the e-coachER package in addition to their usual ERS. In brief, we mailed participants a box containing a user guide to help them access e-coachER, a pedometer and a fridge magnet with tear-off sheets to record weekly step counts or MVPA. The e-coachER web-based support system involved seven ‘Steps to Health’ designed to take about 5–10 min each to complete each week. Participants were not allowed to complete all ‘steps’ in one sitting and were prompted to return each week to enter self-monitored data and get feedback on achievements related to self-identified physical activity goals. We defined getting to step 5 (setting a goal and reviewing a goal online) as a sufficient ‘dose’ of the intervention to impact on minutes of MVPA. For more information, please see the e-coachER logic model in Appendix 1 and the TIDieR checklist which cites the main report [19].

### Outcomes

#### Physical activity

In the interests of consistency and transparency, we chose to examine the mediation effects of process measures on the primary outcome (i.e., a between-group difference in weekly accelerometer recorded MVPA minutes at 12 months) as reported in our trial main findings. MVPA was recorded in ≥ 10 min bouts for the primary outcome using GENEActiv accelerometers (Activinsights; https://www.geneactiv.org/), but we also explored mediation effects on continuous MVPA minutes.^1^ To be included in the analysis for accelerometer measured MVPA, participants had to provide MVPA data recorded over 4 days, including at least one weekend day, for at least 16 h/day. Self-reported MVPA over 1 week was measured using the 7-day recall of PA (7-day Physical Activity Recall questionnaire) at 4 and 12 months [26].

#### Process survey measures

Measures were selected to capture key psychological processes for changing physical activity behaviour, as specified by the underlying logic model (Appendix 1). They reflected theoretical mechanisms of change and enactment (i.e., participant use of the BCTs in day-to-day settings). Briefly, items were derived from extensive reviews of the literature to ensure they matched the theoretical constructs specified by the logic model, but also were fit for purpose within a randomised trial and were acceptable and easy to understand according to our Public and Patient Involvement advisory group.

More details on the rationale for, and selection of, the survey items are provided in Appendix 2, including the construction of multi-item scales to assess the respective constructs. Many existing scales referred to the concept of ‘exercise’ as opposed to ‘physical activity and assumed participants were already active at baseline (e.g., ‘The way I exercise is in agreement with my choices and interests’) [27]. The term ‘exercise’ refers to planned, structured and repetitive activity which is purposeful to improve fitness but since we were interested in changing MVPA more broadly any references to ‘exercise’ were replaced with ‘physical activity’ [28]. Measures were selected based on their brevity, face validity (in the context of a trial in which some participants may find it inappropriate to respond about their beliefs about a behaviour they do not perform), sensitivity to change, content validity (i.e. the extent to which the concepts in the logic model were comprehensively represented by the items in the questionnaire) and internal consistency (the extent to which items measuring the same concept) as outlined by [29]. All process measures were assessed at baseline, and the 4- and 12-month follow-up for the whole sample.

The following process measures were assessed: importance and confidence to be physically active (single item, 11-point scale); perceived competence in being regularly physically active (4 item, 5-point scale); autonomous in decisions about PA (4 items, 5-point scale); availability of support (3 item, 5-point scale); frequency of support (3 item, 5-point scale); action planning (5 item, 5-point scale); and self-monitoring (2 item, 5-point scale). The respective measures were not validated but exploratory factor analysis indicated that Cronbach alpha coefficients of all multi-item scales were over 0.77, using baseline data from participants.

### Analyses

Where accelerometer measured MVPA was the outcome variable, only participants who provided complete accelerometer data at baseline and follow-up and complete data for the process measures were included in analyses. Where self-report MVPA was the outcome variable, only participants who provided valid 7-day recall of PA data and complete data for the process measures were included in analyses. Data cleaning processes have been described elsewhere [19]. For aim 1, between-group differences at 4 and 12 months were examined for each of the eight process measures, using mixed-effects linear models adjusted for age, gender, stratification variables (confidence using IT and reason for referral to ERS), baseline scores for the process variable, and random effects for each recruitment site. These analyses were in line with our pre-specified primary analysis and secondary analysis of other outcomes.

**Fig. 1:**
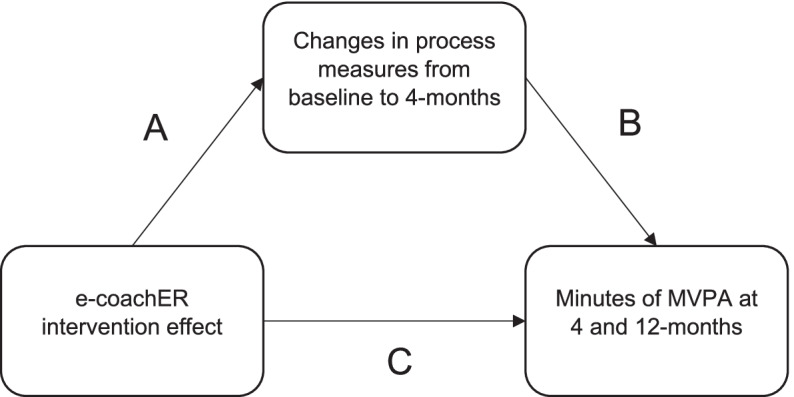
A priori path model for testing mediation effects

For aims 2 and 3, mediation analysis, using the product of coefficient method [30], was conducted to establish the size and significance of any mediating effects for bouted and continuous MVPA. Mediation analysis is still possible and potentially meaningful even when there is no significant effect of the intervention on the primary outcome [31]. This is because lack of effect may reflect a lack of engagement with the intervention processes and so mediation analysis can be used to explain negative as well as positive trial findings. Figure 1 shows a causal diagram with paths of interest. The coefficient, a, for the intervention effect on process measures in path A was derived from the mixed model of changes in process measures regressed on the intervention, adjusted for age, gender, stratification variables baseline scores for the process variable and random effects for each centre. Utilising the same adjustment variables, the coefficient, b, for the change in process measures on the primary outcome in path B was obtained by modelling the outcome on the process measure change, also adjusting for the effect of the intervention. The coefficient of the mediating effect was, therefore, calculated as the product a × b. The confidence intervals were calculated using 1000 bootstrap re-sample iterations. using 1000 bootstrap re-sample iterations as a compromise between the precision of a stable estimate for the bounds of the confidence intervals versus computational time. For missing data, missingness was defined as the absence of data at follow-up for one or more outcomes (i.e., analyses were only conducted if the participant provided data for the particular measures being analysed).

## Results

Table 1 shows the descriptive data for all survey process outcomes at baseline, 4- and 12-months. The process measures were balanced between the two groups at baseline.

**Table 1:** Descriptive data for process measures at baseline and at the 4 and 12-month follow-up

Process measures	Intervention		Control	
	**N**	**Mean (SD)**	**N**	**Mean (SD)**
Importance				
Baseline	96	5.58 (2.58)	121	5.49 (2.90)
Month 4	95	7.55 (2.22)	117	6.53 (2.76)
Month 12	100	7.14 (2.55)	122	6.34 (2.77)
Confidence				
Baseline	97	6.06 (2.73)	121	5.60 (3.10)
Month 4	95	6.72 (2.82)	117	5.56 (3.28)
Month 12	100	6.07 (2.94)	122	5.44 (3.28)
Competence				
Baseline	97	13.74 (3.46)	123	13.14 (3.65)
Month 4	93	14.27 (3.64)	113	12.69 (3.92)
Month 12	99	13.40 (4.09)	118	12.51 (3.94)
Autonomy				
Baseline	98	14.54 (3.18)	121	14.26 (3.48)
Month 4	93	15.31 (3.31)	116	14.69 (3.64)
Month 12	96	15.32 (3.41)	121	14.53 (3.45)
Support availability				
Baseline	97	10.47 (2.93)	122	9.89 (3.39)
Month 4	94	10.80 (2.87)	115	9.77 (3.38)
Month 12	97	10.36 (3.18)	121	9.69 (3.30)
Support frequency				
Baseline	99	7.61 (3.17)	122	7.01 (3.50)
Month 4	94	8.03 (3.41)	116	7.58 (3.62)
Month 12	100	7.70 (3.38)	120	6.97 (3.62)
Use of action planning				
Baseline	97	13.13 (5.03)	117	12.99 (5.25)
Month 4	92	17.09 (4.67)	114	16.10 (5.00)
Month 12	97	15.88 (4.91)	120	14.84 (5.19)
Use of self-monitoring				
Baseline	98	5.70 (1.97)	121	5.17 (2.16)
Month 4	94	7.36 (2.03)	115	6.60 (2.02)
Month 12	99	6.70 (2.09)	121	6.32 (1.95)

*Notes*: Importance and confidence (single item, 11-point scale); competence and autonomy, (4 item, 5-point scale); support availability and support frequency (3 item, 5-point scale); action planning (5 item, 5-point scale); and self-monitoring (2 item, 5-point scale); N varies due to lack of valid wear-time for PA, or non-completion of full set of measures

With respect to the first aim, Table 2 shows the effects of the intervention compared with usual ERS on the process measures from the adjusted mixed-effects linear models. At 4-months, participants in the intervention arm reported significantly more favourable PA beliefs for importance, confidence, competence, availability of support, use of action planning and self-monitoring than participants in the usual ERS arm. At 12-months, participants in the intervention arm reported more favourable PA beliefs for importance than participants in the usual ERS arm.

**Table 2:** The effects of the e-coachER intervention, compared with usual ERS, on process outcomes at 4- and 12-months post randomisation

Process outcomes	N	Coefficient (95% confidence interval)
Importance		
Month 4	204	1.01 (.42 to 1.61)**
Month 12	213	.75 (.05 to 1.45)*
Confidence		
Month 4	205	1.28 (.57 to 1.98)**
Month 12	214	.56 (-.15 to 1.29)
Competence		
Month 4	201	1.61 (.68 to 2.54)**
Month 12	211	.88 (-.13 to 1.89)
Autonomy		
Month 4	203	.70 (-.16 to 1.56)
Month 12	211	.71 (-.16 to 1.58)
Support availability		
Month 4	204	.77 (.07 to 1.48)*
Month 12	211	.39 (-.36 to 1.14)
Support frequency		
Month 4	207	.34 (-.55 to 1.23)
Month 12	215	.51 (-.40 to 1.42)
Use of action planning		
Month 4	196	1.54 (.23 to 2.85)*
Month 12	205	.92 (-.46 to 2.29)
Use of self-monitoring		
Month 4	205	.76 (.19 to 1.32)**
Month 12	213	.31 (-.23 to .85)

*Notes*: *p < .05, **p < .01; N varies due to lack of valid wear-time for PA, or non-completion of full set of measures

For the second aim, there were no direct effects of e-coachER on minutes of accelerometer measured MVPA (recorded in ≥ 10-min bouts) at 12-months when controlling for change in any of the change in process measures at 4-months (c’ -path, Table 3). However, mediation analysis revealed that change in importance, action planning and self-morning at 4-months significantly mediated the effect of e-coachER on minutes of accelerometer measured MVPA (recorded in ≥ 10-min bouts) at 12-months (mediated effect, Table 3). Therefore, despite e-coachER not having a net effect on mean levels of minutes of accelerometer measured MVPA (recorded in ≥ 10-min bouts) at 12-months, it still led to increases in MVPA for some participants via changes in their importance, use of action planning and self-monitoring (Table 3).

**Table 3:** Mediation effects for intervention effects on process outcomes at 4-months on accelerometer measured MVPA (recorded in ≥ 10-min bouts) at 12-months

	**A path**	**B path**	**C’ path**		**Mediated effect**	
**Process measures (N)**	**β (SE)**	**β (SE)**	**β (SE)**	**95%CI**	**β (SE)**	**95%CI**
Importance (204)	1.01 (.30)**	2.48(.92)**	-1.07 (4.80)	-10.48, 8.34	2.52 (1.26)	.45, 5.39
Confidence (205)	1.28 (.36)**	1.43 (.83)	.01 (4.84)	-9.47, 9.49	1.83 (1.25)	-.39, 4.29
Competence (201)	1.61 (.47)**	.27 (.61)	2.41 (4.75)	-6.91, 11.72	.43 (1.29)	-2.18, 2.76
Autonomy (203)	.70 (.44)	.44 (.68)	.76 (4.83)	-8.72, 10.23	.31 (.54)	-.68, 1.58
Support availability (204)	.77 (.36)*	-.10 (.82)	2.99 (4.66)	-6.15, 12.13	-.08 (.71)	-1.60, 1.34
Support frequency (207)	.34 (.46)	1.57 (.60)**	2.83 (4.50)	-6.00, 11.65	.53 (.77)	-1.0, 2.28
Action Planning (196)	1.54 (.67)*	1.01 (.36)**	1.00 (4.69)	-7.01, 10.71	1.56 (.89)	.10,3.54
Self-monitoring (205)	.76 (.29)**	2.53 (.83)**	1.85 (4.52)	-8.19, 10.19	1.92 (1.06)	.21, 4.33

*Notes*: *p < .05, **p < .01; N varies due to lack of valid wear-time for PA, or non-completion of full set of measures

For the third aim, exploratory mediation analysis, there were no direct effects of e-coachER on minutes of accelerometer measured continuous MVPA or self-reported MVPA at 12-months when controlling for change in any of the change in process measures at 4-months. An increase in action planning at 4-months mediated intervention effects on minutes of continuous accelerometer recorded MVPA at 12-months (β = 6.20, 95% CI 0.37 to 14.14). For self-reported PA intervention effects on importance (β = 15.01, 95% CI 1.77 to 30.84), confidence (β = 25.94, 95% CI 4.44 to 52.09), competence (β = 39.73, 95% CI 12.25 to 70.64) and self-monitoring (β = 10.75, 95% CI 1.03 to 24.74) at 4-months mediated intervention effects on minutes of self-reported PA at 4-months. However, only increases in competence at 4-months mediated intervention effects on minutes of self-reported PA at 12-months (β = 17.82, 95% CI 1.83 to 37.53). There were no mediation effects on continuous MVPA at 12 months or on bouted or continuous MVPA at 4-months. See Appendix 3, Tables 1–5 for the full analyses.

## Discussion

The present analysis indicates that most of the processes targeted by e-coachER (apart from autonomy and frequency of support) increased at 4-months over and above ERS alone. We also found that e-coachER increased MVPA for some participants via changes in their importance, use of action planning and self-monitoring.

These findings partially support a recent meta-analysis that showed that interventions informed by SDT were successful in improving competence (g = 0.31) but not relatedness. However, this review also found increases in autonomy (g = 0.37) which contradicts our findings [32]. The lack of change in autonomy and frequency of support could be because ERS professionals were already targeting these processes across the three recruitment sites, meaning e-coachER had no additional effect. The study that found no intervention effects involved adding an SDT-based intervention to standard cardiac rehabilitation [33] and the authors concluded that the (4-week) intervention may have been too limited to create any appreciable augmentation effect. Duda and colleagues trained practitioners to increase their autonomous support, relative to usual ERS support and were also unable to show an augmented effect [9], which the authors attributed to a possible lack of intervention delivery fidelity. The present study involved a digital approach to augmenting usual ERS, so delivery fidelity was an unlikely reason for a lack of an augmented effect. Without a passive control group in each of these augmentation studies, it is difficult to fully interpret the findings, as augmentation interventions will likely be most effective when the comparison group provides no autonomous support. Future studies should therefore seek to compare augmented interventions with interventions involving different levels of existing autonomous support.

Another one of the reasons for the lack of change in autonomy and only partial change in relatedness could be that BCTs used in e-coachER were disproportionally weighted towards increasing competence over relatedness and autonomy. A recent study that mapped BCTs onto constructs of SDT [34] suggests that the BCTs used in e-coachER (self-monitoring of behaviour, goal setting (behaviour), action planning, and review behavioural goals) are all focused on increasing competence. Based on the work by Teixeira and colleagues (2020), the only clear BCT for promoting relatedness was social support, and there were no distinct BCTs for promoting autonomy [13]. This could be because BCTs fostering competence tend to be more practical and distinct (e.g. prompting someone to set a goal) whereas BCTs fostering autonomy tend to be more nuanced and holistic (e.g. using non-controlling language) making it harder to operationalise in a web-based intervention.

A further possible reason for the lack of difference in groups on some of the process outcomes could be a lack of design fidelity, meaning that the BCTs may have not fully operationalised the theoretical constructs as intended [35]. Design fidelity refers to the extent to which self-delivered interventions/intervention protocols reflect their underlying BCTs as intended [11, 35]. The e-coachER intervention used BCTs to operationalise the logic model in the final web-based intervention which was reviewed by the study team. However, e-coachER did not adopt a systematic, unbiased process to ensure that each BCT was adequately operationalised. As such, certain BCTs may have been more salient in the final intervention than others.

Self-monitoring is an effective process for increasing MVPA (e.g., Harris et al., 2017) [36] but little is known about the most effective way to enhance this process. NICE guidelines recommend that ERS monitor a person's progress, provide feedback, agree on goals and develop action plans to help change behaviour [37]. As activity tracking technology such as pedometers and smartphone apps have become readily available, usual ERS support possibly involves guidance on using such devices outside structured exercise environments. Despite this, the e-coachER trial showed an augmented effect of self-monitoring and action planning.

Several of the processes mediated intervention effects on either accelerometer measured MVPA (i.e., importance, action planning and self-monitoring) or self-reported MVPA (competence) at 12-months. Techniques consistent with behavioural regulation (i.e., self-monitoring and action planning) but not beliefs about capabilities (i.e., confidence and competence) were found to mediate intervention effects on accelerometer measured MVPA at 12-months. These findings contrast with a recent systemic review of 51 studies which found that, on average, intervention effects on PA were significantly mediated by beliefs about capabilities, but not behavioural regulation [22]. In contrast, we found that an increase in competence mediated intervention effects on self-reported PA at 12-months. This aligns with the systematic review where, indeed, most of the included studies used self-reported measures of PA rather than device measured PA. Furthermore, the systematic review found generally small mediation effects across all constructs and included a heterogeneous sample of studies across different populations, interventions, and study designs.

### Strengths and limitations

This study had many strengths. First, this is one of the first studies to look at intervention mediation effects on accelerometer measured physical activity at 12-months within a randomized controlled trial (RCT) on a clinical population. A recent systemic review included 51 studies that evaluated mediators of physical activity behaviour change interventions in adults [22]. Of these studies, only three measured physical activity using accelerometers and only three followed up participants after one year. Only one of these studies explored mediation effects on accelerometer recorded physical activity up to one year [38]. Second, the use of bootstrapping accounted for the non-normality in the distribution of the product of two coefficients. Third, we measured participant enactment of two of the core e-coachER BCTs (action planning and self-monitoring) providing a measure of ‘effective engagement’. Effective engagement is defined as sufficient engagement with the intervention to achieve intended outcomes and is rarely measured in behavioural interventions promoting physical activity [12, 39]. Fourth, the use of BCTs which were mapped onto the theoretical underpinnings of SDT (something which is not consistently done in behavioural interventions) [40].

This study also had some limitations. First, the measures for capturing key process measures relating to SDT had to be adapted for e-coachER as they referred to 'exercise', a term that the e-coachER intervention was actively trying to avoid. As such, we took the pragmatic decision to reword scale items to capture physical activity. However, whilst increasing face validity, the measure may have compromised other psychometric properties. Second, the study lacked the full range of measures capturing SDT and enactment, meaning that other important processes may have been missed. However, a large battery of measures was already employed to collect primary and secondary outcomes, so we decided to keep the process evaluation brief to promote engagement and reduce participant burden. Furthermore, we ensured we prioritised the key process measures that we deemed to underpin the e-coachER logic model. Third, the RCT was powered to detect between-group changes in minutes of accelerometer measured MVPA (recorded in ≥ 10-min bouts) at 12-months, not mediated effects. Therefore, the results should be interpreted with some caution.

### Implications

These findings offer important insights for the design and implementation of web-based interventions within existing healthcare contexts. The findings suggest that adding web-based interventions into existing contexts that already provide some degree of face-to-face support might be enough to instigate changes in important modifiable determinants of behaviour change. However, web-based interventions alone may not be enough to change all important variables related to SDT, specifically autonomy and relatedness, and a more guided approach might be required. Future research should build on our findings examine the mediation effect on device measured MVPA in an RCT with people with chronic conditions across a range of different contexts. Previous qualitative research has shown that whilst people from different socioeconomic status (SES) share motives for PA (e.g., maintain health, enjoyment, socialisation), they have different barriers to access (e.g., poorer health, safety concerns and financial restrictions) [41]. A recent systematic review found that digital interventions, which often employed BCTs targeting motivation to increase physical activity were effective in people of higher SES. However, there was no evidence that digital interventions were effective in individuals of lower SES [42]. Whilst e-coachER could not change participants’ environmental factors, it was designed to increase motivations to outweigh or overcome these barriers. We were unable to test whether our attempts to change motivational factors were effective or not depending on the existence of specific operational barriers. However, all analyses controlled for age, gender and site and these factors did not moderate the intervention effects on MVPA. When developing the intervention, we were aware that many ERS subsidised patients to attend for those who couldn't afford it. The e-coachER intervention also offered free pedometers, and subsidies to buy appropriate footwear and attend sessions. We tested e-coachER with a wide range of service users and tried to use non-technical language to ensure functionality for those with a wide range of IT literacy. However, given the lack of effects of e-coachER on MVPA, future interventions could take a more participatory approach with people from a range of backgrounds to ensure that intervention features meet the need for people across a range of backgrounds.

## Conclusion

The findings suggest that some of the key constructs of SDT were adequately targeted to lead to uptake and maintenance of MVPA in primary care patients referred to ERS. It also suggests that engagement with e-coachER, led to changes in autotomy, competence, and relatedness over and above ERS alone. Further work should be carried out to understand how best to design and implement theoretically underpinned web-based physical activity promotion interventions within ERS.

## Data Availability

Data are available upon reasonable request. The guarantor (AT) is willing to examine all requests for the deidentified dataset after a period of three years from the date of this publication.

## Appendix 2

### Selecting and using process survey items for the e-coachER trial

#### Background

Self Determination Theory (SDT) posits that all individuals have three basic psychological needs that need to be fulfilled to lead to self-determined motivation. These are a sense of competence, autonomy and relatedness (Ryan & Deci, 2000). The e-coachER intervention aimed to support each of these needs. For example, reference to achieving 150 min of MVPA or 10,000 steps per day was avoided, and SMART goal setting and action planning were encouraged to avoid failure and enhance a sense of competence. Participants were encouraged to set their own physical activity goals and seek support from others to meet those goals and in doing so gain a sense of autonomy and relatedness.

Exercise referral schemes (ERS) may involve an extension of clinical care in the sense that they are prescriptive and controlling. An exercise practitioner may also adopt an expert/dominant position in the relationship with a referred patient, and in some cases, seek to encourage patients to join the leisure centre/gym after the scheme’s referral period ends, rather than explore other opportunities to be physically active which may have fewer barriers to participation. The e-coachER intervention, therefore, aimed to augment usual ERS, through enhancing a sense of competence, control and connection.

Perceived importance has also been reported to be a key determinant of physical activity (REF to add). If PA is not salient then it is unlikely that an individual would prioritise it over other competing interests and activities. If an intervention can raise interest in and salience of physical activity (for whatever reason) then one may expect someone to prioritise and become more physically active. The e-coachER intervention uses a quiz in Step 1 to highlight the health benefits of PA, and in other Steps, additional benefits (e.g., saving money from commuting by bike) are identified.

Similarly, self-monitoring is widely acknowledged as an important process in behaviour change. The e-coachER intervention involves self-monitoring by initially providing a free pedometer and fridge magnet with space to record steps and minutes of MVPA.

### Selection of process outcome measures

Our goal was to identify and use the most appropriate measures to assess the above theoretical constructs and behaviour change processes.

At the time of designing the study, a review [34] highlighted the most used measures used to capture psychological needs in exercise which have shown good psychometric properties. In this Appendix, Table

**Table 4:** Selection of original items used to previously assess Self-determination theory linked constructs, and our assessment of suitability for use in the e-coachER study

Reference	Subscale	Item	Include?	Reason
Basic Psychological Needs in Exercise Scale [27]	Competence	I feel I have made a lot of progress in relation to the goal I want to achieve	No	Not relevant for baseline assessment of competence as no progress would have been made at baseline
		I feel I perform successfully the activities of my exercise program	No	Not relevant for baseline assessment of competence as refers to exercise programme which participant may not have yet started
		I feel exercise is an activity which I do very well	Maybe	Does not refer to an ‘exercise programme’ however may be confusing/irritating to non exercisers
		I am able to meet the requirements of my exercise program	No	Not relevant for baseline assessment of competence as refers to exercise programme which participant may not have yet started
	Autonomy	The way I exercise is in agreement with my choices and interests	No	Not relevant for baseline assumes already exercising
		I feel that the way I exercise is the way I want to	No	Not relevant for baseline assumes already exercising
		I feel that the way I exercise is a true expression of who I am	No	Not relevant for baseline assumes already exercising
		I feel that I have the opportunity to make choices with regard to the way I exercise	Maybe	May not necessarily imply already exercising
	Relatedness	My relationships with the people I exercise with are very friendly	No	Not relevant for baseline assumes already exercising
		I feel I have excellent communication with the people I exercise with	No	Not relevant for baseline assumes already exercising
		My relationships with the people I exercise with are close	No	Not relevant for baseline assumes already exercising
Perceived Competence Scale (PCS) [43]	Competence	I feel confident in my ability to exercise regularly	Yes	Asks a direct question relating to how one might feel about exercising regularly, but does not assume already is
		I now feel capable of exercising regularly	Maybe	Poor face validity for baseline measurement if participants are not already physically active
		I am able to exercise regularly over the long term	Maybe	Asks about ability to maintain exercise over long term
		I am able to meet the challenge of exercising regularly	Maybe	Asks about ability to meet challenge of exercise
Psychological need satisfaction in exercise scale (PNSE) [44]	Competence	I feel that I am able to complete exercises that are personally challenging	No	Not relevant to baseline non exercisers Refers to ‘challenging’ exercise which may not be appropriate for target population
		I feel confident I can do even the most challenging exercises	No	Not relevant to baseline non exercisers Refers to ‘challenging’ exercise which may not be appropriate for target population
		I feel confident in my ability to perform exercises that personally challenge me	Maybe	Talks about confidence and ability for more personal challenges
		I feel capable of completing exercises that are challenging to me	Maybe	Talks about confidence and ability for more personal challenges
		I feel like I am capable of doing even the most challenging exercises	No	Not relevant to baseline non exercisers Refers to ‘challenging’ exercise which may not be appropriate for target population
		I feel good about the way I am able to complete challenging exercises	No	Not relevant to baseline non exercisers Refers to ‘challenging’ exercise which may not be appropriate for target population
	Autonomy	I feel free to exercise in my own way	Maybe	If amended to ‘physical activity’ may not necessarily imply someone is ‘exercising’ already as assume some level of activity even if very minimal
		I feel free to make my own exercise program decisions	Maybe	If amended to ‘physical activity’ may not necessarily imply someone is ‘exercising’ already as assume some level of activity even if very minimal
		I feel like I am in charge of my exercise program decisions	Maybe	If amended to ‘physical activity’ may not necessarily imply someone is ‘exercising’ already as assume some level of activity even if very minimal
		I feel like I have a say in choosing the exercises that I do	Maybe	If amended to ‘physical activity’ may not necessarily imply someone is ‘exercising’ already as assume some level of activity even if very minimal
		I feel free to choose which exercises I participate in	Maybe	If amended to ‘physical activity’ may not necessarily imply someone is ‘exercising’ already as assume some level of activity even if very minimal
		I feel like I am the one who decides what exercises I do	Maybe	If amended to ‘physical activity’ may not necessarily imply someone is ‘exercising’ already as assume some level of activity even if very minimal
	Relatedness	I feel attached to my exercise companions because they accept me for who I am	No	Not relevant for baseline assumes already exercising
		I feel like I share a common bond with people who are important to me when we exercise together	No	-Not relevant for baseline assumes already exercising
		I feel a sense of camaraderie with my exercise companions because we exercise for the same reasons	No	-Not relevant for baseline assumes already exercising
		I feel close to my exercise companions who appreciate how difficult exercise can be	No	-Not relevant for baseline assumes already exercising
		I feel connected to the people who I interact with while we exercise together	No	Not relevant for baseline assumes already exercising
Intrinsic Motivation Inventory [45]	Autonomy	I exercise because I like to rather than because I feel I have to	No	Not relevant to baseline non exercisers
		Exercising is not something I would necessarily choose to do, rather it is something that I feel I ought to do	Maybe	Does not necessarily imply already exercising
		Having to exercise is a bit of a bind but it has to be done	Maybe	Does not necessarily imply already exercising

4 refers to some of these. However, there were limitations in their direct applicability to e-coachER, namely:

- Use of the term ‘exercise’ as opposed to ‘physical activity'. Both terms may have key conceptual differences for users. It was deemed that physical activity was a more inclusive term and could include less vigorous activities which may not be deemed as ‘exercise’ per se (e.g., walking).
- e-coachER targeted people who are not already achieving at least 150 min of MVPA per week. Some of the existing measures assumed that the participant was already active at baseline.

To get around these issues, existing measures were selected primarily based on the ease of changing the behaviour from exercise to physical activity, and their relevance to people who may be sedentary at baseline. If these could not be found, new items were generated.

Measures were selected or adapted based on the following criteria: conciseness, face validity, sensitivity to change and where possible, some evidence of construct validity and internal reliability. Many existing scales referred to the concept of ‘exercise’ as opposed to ‘physical activity’ [27, 44]. Exercise refers to planned, structured and repetitive activity, which is purposeful to improve fitness, while. physical activity on the other hand encompasses daily activities such as active commuting, domestic activities, occupational activity and recreation [46]. e-coachER was designed to broadly increase physical activity, so references in items to exercise were replaced with physical activity. Existing scales also had many items which assume a participant is already active at baseline (e.g. ‘The way I exercise is in agreement with my choices and interests’ taken from [27]. To pick up baseline cognitions about physical activity, items were only selected if they could apply to participants who were sedentary.

In this Appendix, Table

**Table 5:** Theoretic construct and item(s) used for assessing processes at baseline and 4 months

Construct or Process	Measure
PERCEIVED IMPORTANCE	Measure was generated specifically for this study to be sensitive to the concept that someone might not see the importance about the specific recommended guidelines (5 days of at least 30 min) for PA until after exposure to e-coachER **Question used:** Using an 11 point Likert scale (0 = Strongly Disagree, 10 = Strongly Agree) **Doing at least 30 min of moderate intensity physical activity (e.g. brisk walk) on at least 5 days a week is important to me**
PERCEIVED CONFIDENCE	Measure was generated specifically for this study to be sensitive to the concept that someone might not be confident about achieving the specific recommended guidelines (5 days of at least 30 min) for PA until after exposure to e-coachER **Question used:** Using an 11 point Likert scale (0 = Strongly Disagree, 10 = Strongly Agree) **I am confident that I can do at least 30 min of moderate intensity physical activity (e.g. brisk walk) on at least 5 days a week**
COMPETENCE	Perceived competence scale (PCE) used in Williams, Freedman, and Deci (1998) [43] 4 item measure perceived competence for specific activity assuming one is intending to start or maintain said activity. Amended to reflect ‘physical activity’ as opposed to ‘exercise’. Can be taken at any time point as does not assume one is already active at baseline **Questions used:** Using a 5 point scale (1 = strongly disagree; 2 = disagree; 3 = neither agree nor disagree; 4 = agree; 5 = strongly agree). NB. 1 item to recode **I feel confident in my ability to be physically active regularly** **I do not feel capable of being physically active regularly.** *(NB. reverse code (5* = *strongly disagree; 4* = *disagree; 3* = *neither agree nor disagree; 2* = *agree; 1* = *strongly agree)* **I am able to be physically active regularly** **I am able to meet the challenge of being physically active regularly** Scoring instructions: reverse code the one item then sum all 4 items
AUTONOMY	Perceived Autonomy (Taken from the Psychological need satisfaction in exercise scale (PNSE) [44]. 4 item (reduced from 5 items) subscale measure for perceived autonomy. Amended to say ‘physical activity’ as opposed to ‘exercise’. Factor loadings available to reduce items based on strongest correlations **Questions used:** Using a 5 point scale (1 = strongly disagree; 2 = disagree; 3 = neither agree nor disagree; 4 = agree; 5 = strongly agree). NB. 1 item to recode **I feel free to be physically active in my own way** **I feel free to make my own decisions about physical activity** **I feel like I am in charge of how often I do physical activity** **I do not feel free to choose which physical activities I participate in** *(NB. reverse code (5* = *strongly disagree; 4* = *disagree; 3* = *neither agree nor disagree; 2* = *agree; 1* = *strongly agree)* Scoring instructions: reverse code the one item then sum all 4 items
RELATEDNESS (AVAILABILTY OF SUPPORT)	Items asking if there are people in your life who you can be physically active with. Measure developed specifically for this study as many existing measures of relatedness assumed that participants were already exercising at baseline (e.g.” I feel attached to my exercise companions because they accept me for who I am”) **Questions used:** Using a 5 point scale (1 = strongly disagree; 2 = disagree; 3 = neither agree nor disagree; 4 = agree; 5 = strongly agree) **There are others in my life with whom I can be physically active** **There are people in my life I can enjoy being physically active with** **There are people in my life who can encourage me to be physically active** Scoring instructions: Sum all 3 items
RELATEDNESS (FREQUENCY OF SUPPORT)	3 items taken and amended from a previous 5 item 5 point Likert scale showing reliability and validity from a previous study by [47], (alpha = 0.76 at baseline). Only items that did not assume baseline physical activity levels were included **Questions used:** Using a 5 point scale (1 = almost never; 2 = once in a while; 3 = sometimes; 4 = often; 5 = very often) ‘In the last 30 days how often did others…..’ **Discuss physical activity with me?** **Encourage me to do physical activity?** **Share ideas with me on how to get enough physical activity?** Scoring instructions: Sum all 3 items
ACTION PLANNING	Action/Coping planning scale [48]. 5 items using 5 point scale on action planning with all references to ‘exercise’ changed to ‘physical activity’ **Questions used:** Using a 5 point scale (1 = strongly disagree; 2 = disagree; 3 = neither agree nor disagree; 4 = agree; 5 = strongly agree) ‘In the last 30 days I have regularly made weekly plans for…’ **When to be physically active** **Where to be physically active** **How often to be physically active** **What to do if something interferes with my plans** **How to cope with possible setbacks** Scoring instructions: Sum all 5 items
SELF MONITORING	Self-monitoring for physical activity with 2 items taken from [49] and 3 new items taken from NDPS. Any references to ‘exercise’ amended to ‘physical activity’ **Questions used:** Using a 5 point scale (1 = strongly disagree; 2 = disagree; 3 = neither agree nor disagree; 4 = agree; 5 = strongly agree) ‘In the last 30 days I have… **Consistently monitored the amount of physical activity I do** **Regularly thought about how much physical activity I am doing** Scoring instructions: Sum both items

5 shows the construct of interest or behaviour change process, how the associated question(s) were derived, which items were used and how they were scored. All items were assessed at baseline and 4 and 12 months.

## Appendix 3

### Mediation effects for process outcomes on MVPA

Tables

**Table 6:** (Appendix 3 Mediation effects for change in process outcomes at 4-months on on continuously measured MVPA at 12-months

	**A path**	**B path**	**C’ path**		**Mediated effect**	
**Processes (N)**	**β (SE)**	**β (SE)**	**β (SE)**	**95%CI**	**β (SE)**	**95%CI**
Importance (204)	1.01 (.30)**	3.89 (3.81)	-10.94 (19.81)	-49.78, 27.89	3.95 (4.15)	-3.80, 12.55
Confidence (205)	1.28 (.36)**	.88 (3.44)	-7.59 (19.82)	-46.44, 31.27	1.12 (4.77)	-8.14, 10.08
Competence (201)	1.61 (.47)**	1.08 (2.51)	-4.62 (19.61)	-43.05, 33.81	1.74 (4.56)	-7.98, 10.61
Autonomy (203)	.70 (.44)	2.94 (2.72)	-8.46 (19.51)	-46.70, 29.77	2.05 (2.44)	-1.57, 8.01
Support avail (204)	.77 (.36)*	4.34 (3.35)	-1.58 (19.10)	-39.02, 35.87	3.36 (2.92)	-.96, 10.15
Support freq (207)	.34 (.46)	5.10 (2.48)	.72 (18.75)	-36.02, 37.46	1.73 (2.65)	-2.83, 7.86
Action plan (196)	1.54 (.67)*	4.02 (1.49)	-10.13 (19.41)	-38.22, 36.60	6.20 (3.42)	.37, 14.14
Self-monitor (205)	.76 (.29)**	2.03 (3.47)	-.81 (19.09)	-48.17, 27.91	1.53 (2.78)	-3.69, 7.41

*Notes*: **p <* 0.05, ***p <* 0.01; N varies due to lack of valid wear-time for PA, or non-completion of full set of measures

6,

**Table 7:** (Appendix 3). Mediation effects for *change in* process outcomes at 4-months on accelerometer measured MVPA (recorded in ≥ 10-min bouts) at 4-months

	**A path**	**B path**	**C’ path**		**Mediated effect**	
**Processes (N)**	**β (SE)**	**β (SE)**	**β (SE)**	**95%CI**	**β (SE)**	**95%CI**
Importance (189)	1.09 (.31)***	-.07 (1.10)	.24 (5.61)	-10.76, 11.24	-.08 (1.07)	-1.19, 3.34
Confidence (190)	1.27 (.37)**	.18 (.96)	-.21 (5.56)	-11.10, 10.69	.22 (1.32)	-2.38, 3.25
Competence (187)	1.64 (.48)**	1.06 (.73)	-.52 (5.53)	-11.35, 10.32	1.73 (1.49)	-2.05, 4.11
Autonomy (189)	.64 (.45)	1.04 (.78)	.02 (5.50)	-10.77, 10.81	.67 (.73)	-1.11, 1.93
Support avail (190)	.84 (.36)*	-.19 (.98)	.91 (5.44)	-9.75, 11.57	-.16 (.96)	-2.30, 1.74
Support freq (192)	.32 (.48)	1.02 (.71)	1.34 (5.38)	-9.20, 11.88	.33 (.54)	-0.64, 1.69
Action plan (182)	1.59 (.67)*	.30 (.44)	.72 (5.54)	-9.67, 11.53	.48 (.95)	-2.44, 1.46
Self-monitor (191)	.79 (.30)**	1.46 (.99)	.93 (5.41)	-10.14, 11.57	1.15 (1.07)	-0.57, 3.62

*Notes*: **p <* 0.05, ***p <* 0.01, ****p <* 0.001; N varies due to lack of valid wear-time for PA, or non-completion of full set of measures

7,

**Table 8:** (Appendix 3). Mediation effects for change in process outcomes at 4-months on accelerometer measured MVPA at 4-months

	**A path**	**B path**	**C’ path**		**Mediated effect**	
**Processes (N)**	**β (SE)**	**β (SE)**	**β (SE)**	**95%CI**	**β (SE)**	**95%CI**
Importance (189)	1.09 (.31)***	3.60 (4.75)	32.32 (24.25)	-15.22, 79.85	3.92 (4.58)	-5.98, 12.69
Confidence (190)	1.27 (.37)**	5.68 (4.17)	29.79 (24.03)	-17.31, 76.90	7.23 (5.62)	-3.17, 19.47
Competence (187)	1.64 (.48)**	6.29 (3.14)*	30.34 (23.89)	-16.48, 77.16	10.32 (6.67)	-2.57, 23.65
Autonomy (189)	.64 (.45)	5.55 (3.39)	36.45 (23.82)	-10.22, 83.13	3.54 (3.11)	-1.85, 10.61
Support avail (190)	.84 (.36)*	4.37 (4.19)	40.92 (23.32)	-4.79, 86.62	3.69 (3.63)	-3.51, 11.19
Support freq (192)	.32 (.48)	4.08 (3.05)	38.63 (23.16)	-6.76, 84.01	1.32 (2.30)	-2.97, 6.48
Action plan (182)	1.59 (.67)*	3.08 (1.91)	36.19 (24.26)	-8.51, 82.74	4.90 (3.55)	-2.69, 11.41
Self-monitor (191)	.79 (.30)**	5.99 (4.26)	37.11 (23.28)	-11.35, 83.74	4.72 (3.59)	-1.76, 12.54

*Notes*: **p <* 0.05, ***p <* 0.01, ****p <* 0.001; N varies due to lack of valid wear-time for PA, or non-completion of full set of measures

8,

**Table 9:** (Appendix 3). Mediation effects for *change in* process outcomes at 4-months on self-reported MVPA at 4-months

	**A path**	**B path**	**C’ path**		**Mediated effect**	
**Processes (N)**	**β (SE)**	**β (SE)**	**β (SE)**	**95%CI**	**β (SE)**	**95%CI**
Importance (297)	.68 (.25)**	21.97 (9.03)*	-3.30 (47.15)	-95.71, 89.11	15.01 (7.99)	1.77, 30.84
Confidence (296)	.68 (.30)*	38.26 (7.96)***	-14.71 (46.00)	-104.86, 75.44	25.94 (12.08)	4.44, 52.09
Competence (289)	1.14 (.39)**	34.79 (6.25)***	-20.31 (46.56)	-111.58, 70.95	39.73 (14.58)	12.25, 70.64
Autonomy (291)	.48 (.36)	22.29 (6.92)**	6.60 (47.90)	-87.28, 100.48	10.61 (8.63)	-4.20, 29.55
Support avail (303)	.18 (.38)	3.51 (6.09)	14.59 (47.16)	-77.85, 107.03	.64 (2.41)	-4.28, 6.46
Support freq (295)	.39 (.30)	13.07 (8.59)	10.58 (48.14)	-83.76, 104.92	5.15 (5.00)	-2.86, 16.72
Action plan (288)	.80 (.55)	9.83 (3.78)**	-5.87 (48.87)	-101.66, 89.92	7.91 (5.87)	-2.58, 21.46
Self-monitor (298)	.70 (.23)**	15.42 (8.68)	7.01 (47.66)	-86.40, 100.42	10.75 (6.14)	1.03, 24.74

*Notes*: **p <* 0.05, ***p <* 0.01, ****p <* 0.001; N varies due to non-completion of full set of measures

9,

**Table 10:** (Appendix 3 Mediation effects for change in process outcomes at 4-months on self-reported MVPA at 12-months

	**A path**	**B path**	**C’ path**		**Mediated effect**	
**Processes (N)**	**β (SE)**	**β (SE)**	**β (SE)**	**95%CI**	**β (SE)**	**95%CI**
Importance (268)	.68 (0.25)**	-.62 (9.69)	61.12 (51.47)	-39.75, 161.99	-.42 (8.49)	-19.95, 15.43
Confidence (268)	.68 (0.30)*	15.13 (8.62)	42.50 (50.48)	-56.44, 141.44	10.26 (8.13)	-4.37, 27.26
Competence (262)	1.14 (0.39)**	15.61 (6.16)	22.23 (46.47)	-68.86, 113.31	17.82 (9.30)	1.83, 37.53
Autonomy (264)	.48 (0.36)	16.21 (7.34)	60.72 (51.15)	-39.53, 160.97	7.72 (6.54)	-3.62, 23.64
Support freq (274)	.18 (0.38)	4.87 (6.59)	60.60 (50.02)	-37.44, 158.64	.88 (3.10)	-4.69, 8.07
Support avail (268)	.39 (0.30)	13.59 (9.02)	49.61 (50.70)	-49.76, 148.98	5.35 (5.53)	-3.00, 18.57
Action plan (258)	.80 (0.55)	7.77 (4.03)	34.72 (52.31)	-67.80, 137.24	6.25 (5.59)	-2.65, 18.93
Self-monitor (270)	.70 (0.23)**	4.05 (8.20)	41.99 (45.30)	-46.79, 130.77	2.82 (6.72)	-10.24, 17.07

*Notes*: **p <* 0.05, ***p <* 0.01, ****p <* 0.001; N varies due to non-completion of full set of measures.

10

## Abbreviations

ERS: Exercise referral schemes
PA: Physical activity
BCTs: Behavior change techniques
SDT: Self-determination theory
MVPA: Moderate to vigorous intensity physical activity
GPPAQ: General Practice Physical Activity Questionnaire
SES: Socioeconomic status
RCT: Randomized controlled trial

## Footnotes

1 During the course of the trial the UK guidelines for physical activity were revised and the recommendation that PA needs to be accumulated in 10 min bouts was removed.
